# Neurogenic bladder: etiology and assessment

**DOI:** 10.1007/s00467-008-0764-7

**Published:** 2008-04-01

**Authors:** Stuart B. Bauer

**Affiliations:** 1 Children’s Hospital Boston, Harvard Medical School, Boston, MA USA; 2 Department of Urology, Children’s Hospital Boston, 300 Longwood Avenue, Boston, MA 02115 USA

**Keywords:** Pediatric neurogenic bladder, Myelodysplasia, Diagnosis and evaluation

## Abstract

A review of the various causes of neurologic impairment to the lower urinary tract in children was the aim of this presentation. The emphasis was on diagnosis, pathophysiology, and treatment that strive to maintain as normal a function as possible in order to achieve eventual urinary continence and health of the upper urinary tract. The latest principles based on the most up to date evidence are promulgated but with an eye towards historical prospective. The reader should gain an adequate understanding of various disorders that comprise this condition and feel comfortable with proposing options for management when faced with the responsibility of caring for an affected child.

## Introduction

Neurogenic bladder dysfunction in children is an ever-evolving condition. The expansion of its understanding and treatment over the past 50 years has been just remarkable. In the mid 1950s there were few insights and minimal alternatives to the child’s being in diapers or wearing an appliance over an abdominal wall stoma. Starting with the development of adequate X-ray assessment and reliable urodynamic investigation, the advent of clean intermittent catheterization, artificial sphincter implantation, continent urinary conduits and a plethora of drug therapies that modulate lower urinary tract function, we have learned a great deal about the pathophysiology, pathogenesis and treatment of this disorder and the evidence specific ways to manage it. With the promise of tissue engineering and stem cell therapy, new vistas for treatment seem to be on the horizon.

The most common cause of neurogenic bladder dysfunction in children is neurospinal dysraphism, primarily an open back lesion, but an occult or closed dysraphic state is being diagnosed with more frequency as neonatal spinal ultrasound and magnetic resonance imaging (MRI) are used with increasing regularity to visualize any lower midline spinal cutaneous or gluteal cleft malformation. It was thought that folic acid deficiency was a cause of this disease and that its replacement in women of childbearing age would practically eliminate the condition, but this has not happened. There has been a definite decrease in its incidence; however, in parts of the USA its prevalence has not diminished at all [1–4].

Other causes of neurogenic dysfunction involving the spine include sacral agenesis, tethered spinal cord associated with imperforate anus, cloacal malformations, and spinal cord injuries from sporting injuries and motor vehicle accidents. Central nervous system abnormalities include spastic diplegia (cerebral palsy) and learning disabilities, i.e. attention deficit hyperactivity disorder (ADHD) or attention deficit disorder (ADD). Terminology used throughout this manuscript will conform to the standardization document recently published in the *Journal of Urology* [5].

## Urodynamic studies

A urodynamic study consists of the following components. The child is catheterized with a triple-lumen urodynamic catheter after a small amount of liquid lidocaine (1%) has been injected into the urethra and held in place for a moment or two. First, intravesical pressure is recorded; then, the bladder is drained and the residual urine carefully measured, yielding a pressure at residual volume (this helps determine detrusor compliance at natural filling and is more accurate than cystometric compliance measured during even slow filling of the bladder). A small balloon catheter is passed into the rectum to measure intra-abdominal pressure during the cystometrogram to identify artifacts of motion and monitor increases in abdominal pressure during the filling and emptying phases of the study. The side-hole port of the urethral pressure channel is positioned at the highest point of resistance in the urethra and kept in place, measuring this resistance throughout bladder filling and emptying to determine the leak point pressure. External urethral sphincter electromyography (EMG) is performed using a 24-gauge concentric needle electrode inserted perineally in boys or para-urethrally in girls and advanced into the skeletal muscle component of the sphincter until individual motor unit action potentials are seen or heard on a standard EMG recorder. The characteristics of the individual motor unit potentials at rest, in response to various sacral reflexes (i.e. bulbocavernosus, anocutaneous, Valsalva and Credé maneuvers) and bladder filling and emptying are recorded to detect degrees of denervation. Next, the bladder is filled through the second port while intravesical pressure is monitored via the third port of the tri-lumen urodynamic catheter. The rate of filling is set at 10% of expected capacity for age [age (in years) +30 × 30 = expected capacity in milliliters] [6]. Detrusor pressure measurements are continuously recorded throughout filling to calculate compliance, and during voiding or leaking to denote emptying pressure. Detrusor overactivity is defined as any short-lived pressure rise of >15 cm H_2_O from baseline before capacity is reached [6]. Sometimes, the urodynamics study is combined with fluoroscopic video-imaging using a dilute radio-opaque contrast agent to visualize the appearance of the bladder wall and bladder neck or to detect the presence of vesicoureteral reflux during the test. Alternatively, a radionuclide agent is instilled, with the patient lying above a nuclear camera, to determine at what pressure reflux occurs, when it is known to be present beforehand. The study is not considered complete until the child actually urinates or leaks and the ‘voiding’ pressure is measured. The small size of the urodynamic catheter does not seem to affect the voiding pressure adversely, even in very young children. The normal end filling pressure should be <10 cm H_2_O, while the normal voiding pressure varies from 55 cm to 80 cm H_2_O in boys and from 30 cm to 65 cm H_2_O in girls [5]. Detrusor overactivity is considered an abnormal finding at any time [6].

The examination findings are considered normal when there is an appropriate capacity, good compliant bladder, with no overactivity, and normal innervation of the sphincter with normal sacral reflexes, and an increase in sphincter activity during filling and complete silencing during emptying. An upper motor neuron lesion is present when there is detrusor overactivity and/or hyperactive EMG responses to sacral reflexes and/or a failure of the sphincter muscle, on EMG, to relax (either partially or completely) with a bladder contraction or leaking at capacity. A lower motor neuron lesion is noted when there are no contractions of the detrusor muscle and/or there is a degree of denervation, either partial or complete, in the sphincter muscle, with characteristic EMG changes in the motor units or no motor unit activity at all, respectively, and little or no response in the sphincter to sacral reflexes and/or bladder filling or emptying [7].

## Neurospinal dysraphism

### Myelomeningocele

With regard to embryology, the developing spinal canal begins on the 18th day of gestation and is completed by day 35, closing in a caudad direction from the cephalic end of the body. Failure of mesodermal in-growth over the developing spinal cord results in an open lesion, most commonly seen in the lumbo-sacral area and, with decreasing regularity, in the thoracic and cervical areas (Table 1). The exposed spinal cord and its nerve roots, some of which may protrude into the meningocele sac, and tension on the spinal cord as the cord ‘rises up’ the canal with elongation of the fetus (from L2, L3 in mid- to late fetal life, to L1 at birth), contribute to a variable picture of neural injury to the lower urinary tract and lower extremities [8]. Coupled with obstruction of the aqueduct to the fourth ventricle (Chiari malformation), with possible herniation of the brainstem and the center for micturition coordination (the pontine mesencephalic center), additional layers of dysfunction are added to those nerve pathways already affected.

**Table 1:** Spinal bony level of myelomeningocele (uppermost vertebral abnormality)

Location	Incidence (%)
Cervical-high thoracic	2
Low thoracic	5
Lumbar	26
Lumbosacral	47
Sacral	20

The over-riding issue at birth is whether or not the child has detrusor external urethral sphincter dyssynergy and whether the infant can empty the bladder completely at low pressure. All newborns with radiologic abnormalities at birth (5–10% with hydronephrosis, reflux) have active obstruction of the bladder outlet that had caused these changes to occur in utero [9]. The presence of elevated detrusor filling pressure, bladder sphincter dyssynergy or high voiding or leaking pressures (above 40 cm H_2_O) at capacity, which can result in upper urinary tract deterioration in as many as 63% of children [9], warrants early intervention with clean intermittent catheterization (CIC) and anticholinergic drugs [10–13]. Thus, the investigation of these children when newborn includes a renal and bladder ultrasound, a catheterized measurement of urine residual after voiding or leaking, a determination of serum creatinine concentration after 7 days of life and a urodynamic study that incorporates both detrusor pressure measurements and urethral sphincter electromyography. Voiding cystography is undertaken when hydronephrosis is present and/or urodynamic studies indicate bladder outlet obstruction with either increased pressure at capacity or bladder sphincter dyssynergy. The incidence of reflux when there is functional obstruction of the bladder outlet can range as high as 50% [14, 15].

Although some clinicians still preach watchful waiting if the renal ultrasound findings are normal, and do not recommend urodynamic studies in the newborn period, opting instead for instituting CIC and drug therapy only with the first sign of ureteral or renal pelvic dilation [16, 17], most centers within the USA now advocate full investigation of the lower urinary tract and initiate prophylactic treatment if there are signs of outlet obstruction and/or elevated bladder filling or voiding pressure [18–20]. The incidence of urinary tract deterioration can be greater than 50% [11, 12, 20] when children with the potential for deterioration are followed with expectant and not preemptive therapy. Even though the ‘watchful waiters’ demonstrate that they can reduce the presence of hydronephrosis and, possibly reflux, the changes in detrusor dynamics are not as easily reversed, and the need for subsequent aggressive management of the bladder to control incontinence and a poorly compliant bladder is commonplace (Table 2). Therefore, CIC is begun when detrusor sphincter dyssynergy, elevated leak point pressures greater than 40 cm H_2_O, and/or reflux grade 3 or higher (on a scale of 1 to 5) are present. Instituting CIC and anticholinergic therapy in infancy has revealed many advantages over time [19–21]: the parents and the child adapt to the routine of CIC much easier than they would have if it were to be begun when the child is older; the bladder often remains very compliant, expanding as the child grows and maintaining appropriate wall thickness as noted on bladder echography; hydronephrosis and vesicoureteral reflux develop in fewer than 10%; continence is readily achieved in greater than 50% with no additional maneuvers; and the need for augmentation cystoplasty to maintain a reasonable organ for storage is markedly reduced from almost 60% to 16% when compared with that in children followed expectantly [22].

**Table 2:** Surveillance in infants with myelodysplasia (until age 5 years) (*IVP* intravenous pyelogram, *ECHO* sonogram, *UDS* urodynamic study, *VCUG* voiding cystourethrogram, *RNC* radionuclide cystogram)

Sphincter activity	Recommended tests	Frequency
Intact-synergic	Post-void residual volume	Every 4 months
	IVP or renal ECHO	Every 12 months
	UDS	Every 12 months
Intact-dyssynergic^a^	IVP or renal ECHO	Every 12 months
	UDS	Every 12 months
	VCUG or RNC^b^	Every 12 months
Partial denervation	Post-void residual volume	Every 4 months
	IVP or renal ECHO	Every 12 months
	UDS^c^	Every 12 months
	VCUG or RNC^b^	Every 12 months
Complete denervation	Post-void residual volume	Every 6 months
	Renal ECHO	Every 12 months

^a^Patients receiving intermittent catheterization and anticholinergic agents

^b^If detrusor hypertonicity or reflux is already present

^c^Depending on degree of denervation

When vesicoureteral reflux is present, CIC effectively lowers the intravesical emptying pressure when the bladder is drained. In addition, anticholinergic medication can be added to lower detrusor filling pressure, increasing compliance without fear of causing urinary retention when combined with CIC. The lowered filling and emptying pressures has proven to be very beneficial; in 30–50% of children reflux is resolved within 2–3 years of its discovery and initiation of therapy.

The disadvantages are few and include a higher rate of bacteriuria (60–70% versus 30%) but a lower rate of symptomatic urinary tract infection (20% versus 40%) during childhood than in children followed expectantly [23, 24]. Because the subsequent risk of reflux is lower, the effect on renal function and the development of scarring is reduced when these prophylactically treated children are compared to those monitored with watchful observation [16, 25].

Credé voiding is not an efficacious form of bladder emptying in children with myelodysplasia, especially if the urethral sphincter is partially or fully innervated. Because most children have intact motor function above L1, any increase in abdominal pressure from a Credé maneuver can lead to a reflexive increase in urethral sphincter activity, thus producing an increase in bladder outlet resistance resulting in “high voiding pressure”. This can be particularly noxious in children with moderate or severe grades of reflux. In addition, as the child grows, the bladder resides more in the pelvis and not intra-abdominally, further reducing the effectiveness of the Credé maneuver.

The key to a stable bladder and, consequently, renal function is the maintenance of a good capacity, highly compliant, detrusor muscle, combined with periodic and complete emptying of the bladder at low pressure. Anticholinergic medication (primarily oxybutynin, tolterodine, glycopyrrolate, hyoscyamine or trospium) and CIC achieve that in a majority of children and provide an added benefit of continence if the child has reasonable bladder outlet resistance [9]. Several alpha sympathomimetic agents, such as phenylpropanolamine, ephedrine or pseudoephedrine, are used to increase bladder outlet resistance when it is not sufficient to maintain continence between CICs. A variety of surgeries has been devised when these conditions cannot be met. A detailed description of the surgical treatment is beyond the scope of this discussion, but, suffice it to say, bowel augmentation onto the bladder has been a common practice to lower pressure and increase capacity in order to make the bladder a useful organ for storage. Disadvantages abound, including problematic mucus production, recurrent urinary infection, electrolyte imbalance, stone formation and the recently documented risk for the late occurrence of cancer in the augmented segment [26].

A plethora of surgical procedures has been designed to increase bladder outlet resistance in those children with a level that is insufficient to maintain continence between catheterizations, that include implantation of an artificial urinary sphincter, bladder neck tightening, using adjacent tissue, a fascial sling, and various bulking agents. All can provide more resistance, but no one procedure is ideally suited for every patient.

Creating a catheterizable urinary stoma has become fashionable in those children with intractable urethral incontinence (with obliteration of the bladder neck) or inability to catheterize their urethra easily due to obesity, poor eye–hand coordination or caretaker issues surrounding genital organ privacy. Long-term success has been achieved that provides the individual with a degree of independence, but problems with stomal stenosis can occur.

## Occult spinal dysraphism

Occult spinal dysraphism has been diagnosed with increasing frequency since the advent of spinal ultrasound and MR imaging [27]. An intraspinal lipoma or lipomeningocele, a diastematomyelia, a fatty filum with tethering, or a dermal sinus tract make up the extent of these disorders. Most pediatricians now will image the back of any newborn with a cutaneous lower midline back lesion, which can be detected in 90% of affected individuals [28]. These lesions include a subcutaneous mass, dermal vascular malformation, hypertrichosis, a midline dimple or sinus tract, a skin tag or an asymmetric gluteal cleft. These lesions often signify an underlying bony and/or spinal cord malformation. Ultrasound within the first 3 months of the infant’s life can easily visualize the intraspinal space. After the infant has reached that age MR imaging is needed to diagnose and/or confirm the presence of a dysraphic state.

Most infants have no other manifestation of this disease (other than the cutaneous lesion) because lower extremity neurologic function is normal. In the past, before intraspinal imaging was feasible, these lesions often went undetected until urinary and/or fecal incontinence became problematic or lower extremity difficulties became evident [28–31]. This frequently occurred around the time of a pubertal growth spurt, when increased traction on the spinal cord took place [32]. As the imaging techniques evolved and urodynamic studies in children were performed at an early age, it became clear that most babies had minimal neurologic impairment at first but that the neurologic lesion progressed with advancing age [33].

The pathophysiology involves apparent tension on the lower end of the spinal cord as the child grows. Normally, the conus medullaris ends at L1, L2 at birth but ‘rises’ cephalad to T12, L1 at puberty. The differential growth rate between the spinal cord and the vertebral bodies stretches the lower cord and cauda equina due to fixation of the filum terminale to the bottom of the vertebral canal, or the nerves roots emanating from the cord become compressed by an expanding intraspinal lipoma. With time, this stretching and/or compression affects the oxidative process of the neural tissue that then leads to impaired function of the lower extremities and/or lower urinary tract [34, 35].

Initial investigation of the affected child includes a urodynamics study and a renal and bladder ultrasound. The findings of urodynamics studies in children less than 1 year old are invariably normal, but when abnormal they are not coupled with abnormalities in the lower extremities [33, 36]. When findings are abnormal, partial denervation in the urethral sphincter muscle or failure of the sphincter to relax during a detrusor contraction are the most common findings in infancy, while extensive denervation of the sphincter and/or an acontractile detrusor combined with changes in lower extremity function are the most common abnormalities in an older child [37]. As noted previously for myelomeningocele in children, a voiding cystourethrogram is warranted only when the urodynamics parameters suggest risk to the upper urinary tract from increased bladder outlet resistance or poor detrusor compliance. Vesicoureteral reflux, hydronephrosis and urinary incontinence are all managed in the same fashion as one would treat children with similar neurologic impairment due to an open spinal abnormality.

The abnormal condition tends to improve following spinal cord de-tethering in the infant, but this normalization is unlikely when an older child is operated on [33, 37–39]. Therefore, specific urologic therapy is not instituted following delineation of an abnormality in infants until urodynamics studies have been repeated 3 months after the condition has been repaired. If the findings have not changed, or the child is older (when the chance that the neurologic abnormality will not improve), treatment based on principles outlined in the section on open spinal lesions is instituted.

Thirty percent of children will have secondary spinal cord tethering over time, some as late as puberty or just beyond it, when the last growth spurt occurs [28, 37, 39]. Therefore, careful surveillance and repeated assessment at the first sign of incontinence or changing lower extremity function is warranted. No child is considered risk free until he or she has reached full adult height.

## Sacral agenesis

Partial or complete absence of the lowermost vertebral bodies is labeled sacral agenesis. The condition can range from the absence of just the last two or three sacral bodies to the absence of sacral and several lumbar bones as well (sirenomyelia). This can be seen in offspring of insulin-dependent diabetic mothers (1%) [40], but it may be part of a genetic disorder due to a deletion of part of chromosome 7 (7q36), leading to absence of an important transcription factor that plays a role in the development of the caudal end of the spinal cord and vertebral column [41]. In familial cases of sacral agenesis associated with the Currarino triad syndrome (presacral mass, sacral agenesis and anorectal malformation), deletions in chromosome 7 (7q) resulting in HLXB9 genetic mutations have been found [42]. A mutation in HLXB9, a homeodomain gene of a 403 amino acid protein, which appears to be responsible for neural plate infolding, has been identified in 20 of 21 patients with familial Currarino triad syndrome and in two of seven sporadic cases of this syndrome [43, 44]. Heterozygote carriers within these families have also been identified [45]. Thus, sacral agenesis may represent one point on a spectrum of abnormalities that encompass a sacral meningocele and ano-rectal malformations [46].

In the newborn period (and even afterwards) these infants appear normal, with no lower extremity abnormality. Unless it is thought of during the examination of a newborn of a diabetic mother, such babies often go undiagnosed. With time, as they have difficulty in toilet training or have urinary infection, it becomes evident that there is a problem [40, 47]. The pathognomic sign is absence of the upper end of the gluteal cleft, with flattened buttocks. When the diagnosis is considered a lateral spine film (or a spinal ultrasound in infants) will confirm the abnormality. A spinal MR reveals a sharp cut off to the cord at about T-12, with nerve roots streaming from it. Approximately 90% of children develop neurogenic bladder dysfunction [48].

An equal number of children have an overactive detrusor with sphincter dyssynergy or an acontractile detrusor with complete denervation in the urethral sphincter [47, 49]. The former is often associated with recurrent urinary infection and vesicoureteral reflux, whereas the latter produces continuous incontinence. The type of neurologic impairment affecting the lower urinary tract cannot be predicted from the level of absent or abnormal vertebral bones [47]. Obviously, management depends on the type of dysfunction present. CIC, anticholinergic medication and antibiotics are instituted in those with an upper motor neuron type lesion, while surgical measures with CIC are needed in those with an incompetent sphincter mechanism.

## Associated conditions

### Imperforate anus

Failure of the lowermost portion of the developing colon in the fetus to canalize fully, resulting in a closed rectum that does not open onto the anal skin verge, is a rare anomaly, but one that needs immediate attention after delivery. In boys there is a fistula from the end of the rectal canal to the posterior urethra, whereas a stenotic, anteriorly placed anal canal that ends at the posterior aspect of the vestibule, may be noted in girls (Table 3). Usually, a colostomy is performed, or, more recently, definitive repair is undertaken in the first 24 h of life in boys, while anal dilatation is begun in girls.

**Table 3:** Wingspread classification of anorectal malformations

Female	Male
High	High
Anorectal agenesis	Anorectal agenesis
With rectovaginal fistula	With rectourethral (prostatic) fistula
Without fistula	Without fistula
Rectal atresia	Rectal atresia
Intermediate	Intermediate
Rectovestibular fistula	Rectovestibular urethral fistula
Rectovaginal fistula	
Anal agenesis without fistula	Anal agenesis without fistula
Low	Low
Anovestibular fistula	Anocutaneous fistula
Anocutaneous fistula	Anal stenosis
Anal stenosis	Rare malformation
Cloacal malformation	
Rare malformation	

From a urologic standpoint, this condition is often part of a constellation of abnormalities known as the VATER or VACTERL association (the mnemonic denotes all affected organs: **V** vertebral; **A** anal; **C** cardiac; **TE** tracheoesophageal fistula; **R** renal; **L** limb). Unilateral renal agenesis, vesicoureteral reflux and spinal cord tethering are the most common abnormalities affecting the urinary tract [50]. Urinary, and fecal, incontinence issues that become prominent as the child matures, due to progressive denervation of the lowermost nerves modulating bladder and urethral and anal muscle function, are likely clinical scenarios. This is most commonly seen in children where the rectum has ended above the levator ani muscle (50%), but it is also seen when the rectum ends below that pelvic floor muscle (18%) [51]. A spinal vertebral bony image is not a reliable sign that spinal cord abnormality is present [52]. A spinal ultrasound in newborns, and MRI of the spine in older children, are a mandatory part of the investigation in these children. An upper motor neuron lesion with detrusor overactivity and/or detrusor sphincter dyssynergy are most likely to develop, but an acontractile detrusor and sphincter denervation are also seen as a result of spinal cord tethering [49, 53]. It has been shown that the earlier neurosurgical intervention is undertaken, the better the individual’s chances of having normal sacral spinal cord and lower urinary tract function [54]. Therefore, early detection of this condition is necessary to improve the child’s chance of maintaining healthy kidneys and becoming continent.

## Central nervous system disorders

### Cerebral palsy

This condition results from a non-progressive injury to the brain, occurring in the perinatal period, that produces a neuromuscular disability or a specific symptom complex of cerebral dysfunction [55]. It is caused by perinatal infection or a period of anoxia (or hypoxia) affecting the tissues of the central nervous system [56] (Table 4). It appears in babies who are less than 2 kg at birth, have had intraventricular hemorrhage, experienced a neonatal seizure or received mechanical ventilation for a prolonged period of time in the postnatal period [57]. The incidence of cerebral palsy is increasing, as more severely premature infants are surviving and going home.

**Table 4:** Perinatal risk factors in cerebral palsy. Adapted from [61] used with permission. *UMN* upper motor neuron lesion, *LMN* lower motor neuron lesion

Factor	UMN (no. of patients)	LMN (no. of patients)
Prematurity	10	1
Respiratory distress/arrest/apnea	9	2
Neonatal seizures	5	–
Infection	5	1
Traumatic birth	5	–
Congenital hydrocephalus	3	–
Placenta previa/abruption	2	2
Hypoglycemia seizures	2	–
Intracranial hemorrhage	2	–
Cyanosis at birth	1	3
No specific factor noted	15	–

Affected children have delayed gross motor development, abnormal fine motor performance, altered muscle tone, abnormal stress gait, tight heel cords and exaggerated deep tendon reflexes [58]. These findings can vary substantially, from being quite obvious to very subtle, unless a careful neurologic examination is performed. These abnormalities may not be manifest in the early postnatal period, but become evident over time, because myelination of axons and maturation of neurons in the basal ganglia are required before spasticity, dystonia, and athetosis become apparent. Some less affected children have even milder forms of the disease, with only learning disabilities, attention deficit, or attention deficit hyperactivity disorders being seen.

Most children with cerebral palsy develop total urinary control, albeit at an age later than most unaffected children. Incontinence is a feature in some (24% of surveyed families in one study) [59], but the exact incidence has never been truly determined. The presence of incontinence is often related to the extent of the physical impairment, primarily because the handicap prevents the child from reaching the bathroom on time, causing an episode of wetting [60]. Urinary infection and vesicoureteral reflux are not features of this disease, and the kidneys are invariably normal on ultrasonic imaging.

An upper motor neuron type of bladder dysfunction with detrusor overactivity (80%), but not necessarily with detrusor sphincter dyssynergy (5%), is invariably present, but lower motor neuron injury with sphincter denervation due to spinal cord involvement can be seen as well (11%) [61] (Tables 5 and 6). Volitional control over sphincter function is present, and affected children have some ability to prevent leaking from an overactive detrusor by tightening the muscle for a variable period of time. Children with milder forms of dysfunction, with just learning disabilities without spasticity, have an overactive detrusor leading to either urgency (with or without incontinence) and nocturia, or day and night wetting. Therefore, modulating the overactivity with anticholinergic medication is the treatment of choice. However, this must be done judiciously, with careful monitoring of residual urine to prevent the development of retention.

**Table 5:** Lower urinary tract function in cerebral palsy

Type	Number
Upper motor neuron lesion	49
Mixed upper + lower motor neuron lesion	5
Incomplete lower motor neuron lesion	1
No urodynamic lesion	2

**Table 6:** Urodynamics findings in cerebral palsy (some patients had more than one finding)

Type of Lesion	No. of Patients
Upper motor neuron (detrusor or sphincter)	
Detrusor overactivity	35
Detrusor sphincter dyssynergy	7
Overactive sacral reflexes	6
No voluntary control	3
Smaller than expected bladder capacity	2
Poorly compliant	2
Lower motor neuron (abnormal motor unit potentials)	
Excessive polyphagia of sphincter	5
↑ Amplitude + ↑ duration potentials	4

## Trauma

### Traumatic injuries to the spine

Fortunately, spinal cord injuries in children are rare (2.6 per million) [62]. The incidence tends to increase geometrically with age [63]. When an injury does occur, it is more likely to happen in a boy than in a girl, and it is usually the result of a motor vehicle or bicycle accident (24–52%), a fall from a high place, a gunshot wound, or a diving or sports incident. Injuries may also occur iatrogenically after surgery to correct scoliosis, kyphosis or other intraspinal processes, congenital aortic anomalies, or patent ductus arteriosus operations [62, 64–67]. Newborns are particularly prone to a hyperextension injury during high forceps delivery [68].

Spinal cord injuries in children are intrinsically different from those in adults, owing to a variety of factors, including the mechanism of injury and the difference in configuration of the cord in children compared with that in adults. In addition, the horizontal versus vertical orientation of the facet joints in vertebral bodies that predisposes to anteroposterior subluxation in children, the delayed supportive effect of the paraspinous musculature and ligaments, and the relative heaviness of the head, which causes a fulcrum of maximal flexion of the upper cervical region in infants and young children, all contribute to a high degree of hypermobility that places the child’s spinal cord at risk for ischemic necrosis [65].

The lower urinary tract dysfunction that ensues is not likely to be an isolated event but is usually associated with loss of sensation and paralysis of the lower limbs. Radiologic investigation of the spine may not reveal any bony abnormality, although momentary subluxation of osseous structures resulting from the elasticity of the vertebral ligaments can result in a neurologic injury. This condition has been seen only in children (usually younger than 8 years old) and has been labeled SCIWORA (***s***pinal ***c***ord ***i***njury ***w***ith***o***ut ***r***adiologic ***a***bnormality) [69]. Overall, SCIWORA can account for up to 38% of spinal cord injuries in children [70]. Often, what appears to be a permanent lesion initially turns out to be a transient phenomenon with time. Although sensation and motor function of the lower extremities may be restored relatively quickly, the dysfunction involving the bladder and rectum may persist considerably longer.

During the acute phase of the injury, the bladder is often acontractile and the urethral sphincter nonreactive, although normal-appearing bioelectric potentials can be recorded on sphincter EMG (spinal shock). Over a variable but unpredictable period of time, detrusor contractility and sphincter reactivity return as spinal cord edema subsides. With this return of function, an overactive detrusor and bladder-sphincter dyssynergy may develop if the lateral reticulospinal cord pathways to and from the brainstem have been disrupted. When the lesion affects the cauda equina, there is probably little to no return of bladder or sphincter function. Sacral sensation and peripheral reflexes are not good indicators of ultimate lower urinary tract function [71]. Over time, the predominant urodynamic pattern in patients with a thoracic-level lesion is an overactive detrusor with sphincter dyssynergy, high voiding pressures, eventual hydronephrosis and vesicoureteral reflux. Patients with an upper thoracic or cervical lesion are likely to exhibit autonomic dysreflexia with a spontaneous discharge of α1 stimulants during bladder filling and with contractions of the detrusor that require careful monitoring of their blood pressure during any investigational studies of the lower urinary tract [72, 73].

Foley catheter draining in the immediate post-injury phase is needed, but CIC should be begun as soon as feasible after that time [74–76]. When a child starts volitional voiding, CIC can be tapered and stopped as residual urines are measured and found to be insignificant (less that 5 ml). Urodynamics studies should be undertaken no earlier than 6 weeks after the injury, to allow for the manifestation of the extent of the neurologic injury [77]. Periodic reassessment of bladder and sphincter function is appropriate up to 2 years after the injury, due to the potential for change during that time. Renal ultrasonography should be part of the assessment, but voiding cystography is only needed when there are signs of potential risk (i.e. sphincter dyssynergy or poor detrusor compliance). Low detrusor filling and voiding pressures with complete emptying are the goal [78]. When this is not present during testing, starting or continuing CIC and adding anticholinergic medication is paramount to insuring the long-term health of the lower and upper urinary tract [79].

## Conclusions

Neurogenic bladder dysfunction in children takes in a very wide spectrum of conditions that include congenitally acquired conditions that may even be preventable today, conditions that are associated with specific anatomic abnormalities, and acquired conditions that may occur perinatally or from accidents or sports or motor vehicle related injuries. Despite the etiology, the guiding principles for management are similar; insuring and maintaining an adequate sized, normally compliant, reservoir that evacuates urine completely, at a relatively low pressure, is the key to maintaining a healthy environment for the kidneys. A plethora of methods has come into existence, especially since the advent of clean intermittent catheterization and the advancement of pharmacologic understanding and manipulation. Future prospects look bright for affected children, with the overall health of the individual the most paramount goal to be achieved.

## Questions

Answers appear following the reference list.

1. Which vitamin plays a major role in the prevention of neural tube defects?
  - Niacin
  - Riboflavin
  - Folic acid
  - B12
  - Carotene
2. What percent of newborns with a myelomeningocele have a normal upper urinary appearance on ultrasound within the first month of life?
  - 45%
  - 60%
  - 75%
  - 90%
  - 98%
3. Credé voiding is best avoided in which of the following conditions?
  - The presence of vesicoureteral reflux with a severely denervated urethral sphincter
  - The presence of vesicoureteral reflux with a fully innervated urethral sphincter
  - A poorly compliant bladder with a low leak point pressure
  - An overactive detrusor with a low leak point pressure
  - A compliant bladder with a low leak point pressure
4. When considering an antireflux operation in a child with a myelomeningocele, treating which urodynamics parameter beforehand is thought to be most important for achieving a successful result?
  - Poor compliance
  - Presence of detrusor overactivity
  - Both conditions noted above
  - Neither condition noted above
  - A high leak point pressure
5. All of the following may be an outward physical sign of an occult spinal dysraphism except
  - A subcutaneous mass overlying the thoracic spine
  - An asymmetric gluteal cleft
  - A draining pilonidal dimple
  - One leg slightly longer than the other
  - Spinal scoliosis
6. The conus medullaris normally resides opposite which vertebral body at puberty?
  - Thoracic 11 level
  - Thoracic 12 level
  - Lumbar 1 level
  - Lumbar 2 level
  - Lumbar 3 level
7. Which of the following maternal factors may be responsible for sacral agenesis in a newborn child?
  - Exposure to progestational agents early in the pregnancy
  - Insulin-dependent diabetes early in the early gestational period
  - Insulin dependency later in the pregnancy
  - Exposure to progestational agents later in the pregnancy
  - None of the above
8. Which of the following statements is most true about sacral agenesis?
  - Motor function in the lower extremities and sacral area are normal
  - Motor function in the lower extremities and sacral area are impaired
  - The abnormality can be diagnosed in the newborn period due to an abnormal gluteal cleft
  - Sensation in the lower extremities and sacral area are normal
  - Sensation in the lower extremities and sacral area are impaired
9. Most children with cerebral palsy have what type of lower urinary tract function on urodynamics testing?
  - A hyperactive bladder with denervation in the external urethral sphincter
  - A hyperactive bladder with bladder sphincter dyssynergy during voiding
  - An underactive bladder with denervation in the external urethral sphincter
  - An underactive bladder with a normally innervated external urethral sphincter
  - A normally reflexic bladder with bladder sphincter synergy during voiding
10. The most common cause of a traumatic spinal cord injury in babies is
  - A motor vehicle accident involving a child who is not wearing a seat belt
  - An inadvertent fall from a high place
  - A hyperextension injury to the cervical spine during delivery
  - Spinal column surgery to correct either an intraspinal process or scoliosis
  - A sports related injury

## References

[1] Palomaki GE, Williams JR, Haddow JE (1999). Prenatal screening for open neural-tube defects in Maine. N Engl J Med.

[2] Centers for Disease Control and Prevention (2004). Spina bifida and anencephaly before and after folic acid mandate—United States, 1995–1996 and 1999–2000. MMWR Morb Mortal Wkly Rep.

[3] Williams LJ, Rasmussen SA, Flores A, Kirby RS, Edmonds LD (2005). Decline in the prevalence of spina bifida and anencephaly by race/ethnicity: 1995–2002. Pediatrics.

[4] Rothenberg SP, da Costa MP, Sequeira JM, Cracco J, Roberts JL, Weedon J, Quadros EV (2004). Autoantibodies against folate receptors in women with a pregnancy complicated by a neural-tube defect. N Engl J Med.

[5] Neveus T, von Gontard A, Hoebeke P, Hjalmas K, Bauer S, Bower W, Jorgensen TM, Rittig S, Vande Walle J, Yeung CK, Djurhuus JC (2006). The standardization of terminology of lower urinary tract function in children and adolescents: report from the standardization committee of the International Children’s Continence Society. J Urol.

[6] Bauer SB, Labib KB, Dieppa RA, Retik AB (1977). Urodynamic evaluation in a boy with myelodysplasia and incontinence. Urology.

[7] Gierup J, Ericsson NO (1970). Micturition studies in infants and children: intravesical pressure, urinary flow and urethral resistance in boys with intravesical obstruction. Scand J Urol Nephrol.

[8] Blaivas JG, Labib KB, Bauer SB, Retik AB (1977). Changing concepts in the urodynamic evaluation of children. J Urol.

[9] Bauer SB (2003) Urodynamics in Myelodysplasia. Bladder and Bowel Dysfunction in Myelodysplasia Symposium, 3 April 2003, Aachen, Germany

[10] McGuire EJ, Wang CC, Usitalo H, Savastano J (1986). Modified pubovaginal sling in girls with myelodysplasia. J Urol.

[11] Bauer SB, Hallet M, Khoshbin S, Lebowitz RL, Winston KR, Gibson S, Colodny AH, Retik AB (1984). The predictive value of urodynamic evaluation in the newborn with myelodysplasia. JAMA.

[12] Sidi AA, Dykstra DD, Gonzalez R (1986). The value of urodynamic testing in the management of neonates with myelodysplasia: a prospective study. J Urol.

[13] Perez LM, Khoury J, Webster GD (1992). The value of urodynamic studies in infants less than one year old with congenital spinal dysraphism. J Urol.

[14] Bauer SB, Johnston JH (1984). Vesico-ureteral reflux in children with neurogenic bladder dysfunction. International perspectives in urology, vol 10.

[15] Seki N, Akazawa K, Senoh K, Kubo S, Tsunoda T, Kimoto Y, Naito S (1999). An analysis of risk factors for upper urinary tract deterioration in patients with myelodysplasia. Br J Urol.

[16] Hopps CV, Kropp KA (2003). Preservation of renal function in children with myelomeningocele managed with basic newborn evaluation and close followup. J Urol.

[17] Teichman JMH, Scherz HC, Kim KD, Cho DH, Packer MG, Kaplan GW (1994). An alternative approach to myelodysplasia management: aggressive observation and prompt intervention. Urol.

[18] Geranoitis E, Koff SA, Enrile B (1988). Prophylactic use of clean intermittent catheterization in treatment of infants and young children with myelomeningocele and neurogenic bladder dysfunction. J Urol.

[19] Edelstein RA, Bauer SB, Kelly MD, Darbey MM, Peters CA, Atala A, Mandell J, Colodny AH, Retik AB (1995). The long-term urologic response of neonates with myelodysplasia treated proactively with intermittent catheterization and anticholinergic therapy. J Urol.

[20] Wu H-Y, Baskin LS, Kogan BA (1997). Neurogenic bladder dysfunction due to myelomeningocele: Neonatal versus childhood treatment. J Urol.

[21] Joseph DB, Bauer SB, Colodny AH, Mandell J, Retik AB (1989). Clean intermittent catheterization in infants with neurogenic bladder. Pediatrics.

[22] Kaefer M, Pabby A, Kelly M, Darbey M, Bauer SB (1999). Improved bladder function after prophylactic treatment of the high risk neurogenic bladder in newborns with myelomeningocele. J Urol.

[23] Cohen RA, Rushton HG, Belman AB, Kass EJ, Majd M, Shaer C (1990). Renal scarring and vesicoureteral reflux in children with myelodysplasia. J Urol.

[24] Schlager TA, Clark M, Anderson S (2001). Effect of a single-use sterile catheter for each void on the frequency of bacteriuria in children with neurogenic bladder on intermittent catheterization for bladder emptying. Pediatrics.

[25] Agarwal SK, McLorie GA, Grewal D, Joyner BD, Bägli DJ, Khoury AE (1997). Urodynamic correlates of resolution of reflux meningomyelocele patients. J Urol.

[26] Metcalfe PD, Rink RC (2007). Bladder augmentation: complications in the pediatric population. Curr Urol Rep.

[27] Bruce DA, Schut L (1979). Spinal lipomas in infancy and childhood. Brain.

[28] Pierre-Kahn A, Zerah M, Renier D, Cinalli G, Sainte-Rose C, Lellouch-Tubiana A, Brunelle F, Le Merrer M, Giudicelli Y, Pichon J, Kleinknecht B, Nataf F (1997). Congenital lumbosacral lipomas. Childs Nerv Syst.

[29] Mandell J, Bauer SB, Hallett M, Khoshbin S, Dyro FM, Colodny AH, Retik AB (1980). Occult spinal dysraphism: a rare but detectable cause of voiding dysfunction. Urol Clin North Am.

[30] Koyanagi I, Iwasaki Y, Hida K, Abe H, Isu T, Akino M (1987). Surgical treatment supposed natural history of the tethered cord with occult spinal dysraphism. Childs Nerv Syst.

[31] Sarica K, Erbagci A, Yagci F, Yurtseven C (2003). Multidisciplinary evaluation of occult spinal dysraphism in 47 children. Scand J Urol Nephrol.

[32] Satar N, Bauer SB, Shefner J, Kelly MD, Darbey MM (1995). The effects of delayed diagnosis and treatment in patients with an occult spinal dysraphism. J Urol.

[33] Keating MA, Rink RC, Bauer SB, Krarup C, Dyro FM, Winston KR, Shillito J, Fischer EG, Retik AB (1988). Neuro-urologic implications of changing approach in management of occult spinal lesions. J Urol.

[34] Yamada S, Won DJ, Yamada SM (2004). Pathophysiology of tethered cord syndrome: correlation with symptomatology. Neurosurg Focus.

[35] Henderson FC, Geddes JF, Vaccaro AR, Berry KJ, Benzel EC (2005). Stretch-associated injury in cervical spondylotic myelopathy. Neurosurgery.

[36] Nogueira M, Greenfield SP, Wan J, Wan J, Santana A, Li V (2004). Tethered cord in children: a clinical classification with urodynamic correlation. J Urol.

[37] Satar N, Bauer SB, Scott RM, Shefner J, Kelly M, Darbey M (1997). Late effects of early surgery on lipoma and lipomeningocele in children less than two years old. J Urol.

[38] Cornette L, Verpoorten C, Lagae L, Van Calenbergh F, Plets C, Vereecken R, Casaer P (1998). Tethered spinal cord in occult spinal dysraphism: timing and outcome of surgical release. Neurology.

[39] Proctor M, Bauer SB, Scott MR (2000). The effect of surgery for the split spinal cord malformation on neurologic and urologic function. Pediatr Neurosurg.

[40] Wilmshurst JM, Kelly R, Borzyskowski M (1999). Presentation and outcome of sacral agenesis: 20 years’ experience. Dev Med Child Neurol.

[41] Papapetrou C, Drummond F, Reardon W, Winter R, Spitz L, Edwards YH (1999). A genetic study of the human T gene and its exclusion as a major candidate gene for sacral agenesis with anorectal atresia. J Med Genet.

[42] Ross AJ, Ruiz-Perez V, Wang Y, Hagan DM, Scherer S, Lynch SA, Lindsay S, Custard E, Belloni E, Wilson DI, Wadey R, Goodman F, Orstavik KH, Monclair T, Robson S, Reardon W, Burn J, Scambler P, Strachan T (1998). A homeobox gene, HLXB9, is the major locus for dominantly inherited sacral agenesis. Nat Genet.

[43] Kochling J, Karbasiyan M, Reis A (2001). Spectrum of mutations and genotype-phenotype analysis in Currarino syndrome. Eur J Hum Genet.

[44] Hagan DM, Ross AJ, Strachan T, Lynch SA, Ruiz-Perez V, Wang YM, Scambler P, Custard E, Reardon W, Hassan S, Nixon P, Papapetrou C, Winter RM, Edwards Y, Morrison K, Barrow M, Cordier-Alex MP, Correia P, Galvin-Parton PA, Gaskill S, Gaskin KJ, Garcia-Minaur S, Gereige R, Hayward R, Homfray T (2000). Mutation analysis and embryonic expression of the HLXB9 Currarino syndrome gene. Am J Hum Genet.

[45] Lynch SA, Wang Y, Strachan T, Burn J, Lindsay S (2000). Autosomal dominant sacral agenesis: Currarino syndrome. J Med Genet.

[46] Bernbeck B, Schurfeld-Furstenberg K, Ketteler K, Kemperdick H, Schroten H (2004). Unilateral pulmonary atresia with total sacral agenesis and other congenital defects. Clin Dysmorphol.

[47] Guzman L, Bauer SB, Hallet M, Khoshbin S, Colodny AH, Retik AB (1983). The evaluation and management of children with sacral agenesis. Urology.

[48] De Biasio P, Ginocchio G, Aicardi G, Ravera G, Venturini PL, Vignolo M (2003). Ossification timing of sacral vertebrae by ultrasound in the mid-second trimester of pregnancy. Prenat Diagn.

[49] Boemers TM, Van Gool JD, deJong TP, Bax KM (1994). Urodynamic evaluation of children with caudal regression syndrome (caudal dysplasia sequence). J Urol.

[50] Parrott TS (1985). Urologic implications of anorectal malformations. Urol Clin North Am.

[51] Shaul DB, Harrison EA (1997). Classification of anorectal malformation: initial approach, diagnostic test, and colostomy. Semin Pediatr Surg.

[52] Rivosecchi M, Lucchetti MC, Zaccara A, De Gennaro M, Fariello G (1995). Spinal dysraphism detected by magnetic resonance imaging in patients with anorectal anomalies: incidence and clinical significance. J Pediatr Surg.

[53] Taskinen S, Valanne L, Rintala R (2002). Effect of spinal cord abnormalities on the function of the lower urinary tract in patients with anorectal abnormalities. J Urol.

[54] Estrada CR, Dokucu A, Borer JG, Khoshbin S, Brisco C, Bauer SB (2006) Early spinal cord untethering and ano-rectal malformation reconstruction does not adversely affect lower urinary tract function. Abstract presentation, Section on Urology, American Academy of Pediatrics, Atlanta, GA, October 9

[55] Kuban KC, Leviton A (1994). Cerebral palsy. N Engl J Med.

[56] Nelson KB, Ellenberg JH (1986). Antecedents of cerebral palsy. N Engl J Med.

[57] Kim JN, Namburg R, Chang W, Oh CH, Shin JC, Park ES, Park CI, Park MS, Park KI, Lee C, Han DG (1999). Prospective evaluation of perinatal risk factors for cerebral palsy and delayed development in high risk infants. Yonsei Med J.

[58] Kyllerman M, Bager B, Bensch J, Bille B, Olow I, Voss H (1982). Dyskinetic cerebral palsy: I. Clinical categories, associated neurological abnormalities and incidences. Acta Paediatr Scand.

[59] Roijen LE, Postema K, Limbeek VJ, Kuppevelt VH (2001). Development of bladder control in children and adolescents with cerebral palsy. Dev Med Child Neurol.

[60] Murphy KP, Molnar GE, Lankasky K (1995). Medical and functional status of adults with cerebral palsy. Dev Med Child Neurol.

[61] Decter RM, Bauer SB, Khoshbin S, Dyro FM, Krarup C, Colodny AH, Retik AB (1987). Urodynamic assessment of children with cerebral palsy. J Urol.

[62] Augutis M, Levi R (2003). Pediatric spinal cord injury in Sweden: incidence, etiology and outcome. Spinal Cord.

[63] Anderson JM, Schutt AH (1980). Spinal injury in children: a review of 156 cases seen from 1950 through 1978. Mayo Clin Proc.

[64] Cass AS, Luxenberg M, Johnson CF, Gleich P (1984). Management of the neurogenic bladder in 413 children. J Urol.

[65] Decter RM, Bauer SB (1993). Urologic management of spinal cord injury in children. Urol Clin North Am.

[66] Batista JE, Bauer SB, Shefner JM, Kelly MD, Darbey MD, Siroky MB (1995). Urodynamic findings in children with spinal cord ischemia. J Urol.

[67] Cirak B, Ziegfeld S, Knight VM, Chang D, Avellino AM, Paidas CN (2004). Spinal cord injuries in children. J Pediatr Surg.

[68] Lanska MJ, Roessmann U, Wiznitzer M (1990). Magnetic resonance imaging in cervical cord birth injury. Pediatrics.

[69] Pang D, Pollack IF (1989). Spinal cord injury without radiographic abnormalities in children: the SCIWORA syndrome. J Trauma.

[70] Brown RL, Brunn MA, Garcia VF (2001). Cervical spine injuries in children: a review of 103 patients treated consecutively at a level 1 pediatric trauma center. J Pediatr Surg.

[71] Shenot PJ, Rivas DA, Watanabe T, Chancellor MB (1998). Early predictors of bladder recovery and urodynamics after spinal cord injury. Neurourol Urodyn.

[72] Vaidyanathan S, Soni BM, Sett P, Watt JW, Oo T, Bingley J (1998). Pathophysiology of autonomic dysreflexia: long-term treatment with terazosin in adult and pediatric spinal cord injury patients manifesting recurrent dysreflexic episodes. Spinal Cord.

[73] Perkash I (1997). Autonomic dysreflexia and detrusor-sphincter dyssynergia in spinal cord injury patients. J Spinal Cord Med.

[74] Guttmann L, Frankel H (1966). The value of intermittent catheterization in the early management of traumatic paraplegia and tetraplegia. Paraplegia.

[75] Barkin M, Dolfin D, Herschorn S, Bharatwal N, Comisarow R (1983). The urologic care of the spinal cord injury patient. J Urol.

[76] Giannantoni A, Scivoletti G, Di Stasi SM, Silecchia A, Finazzi-Agrò E, Micali I, Castellano V (1998). Clean intermittent catheterization and prevention of renal disease in spinal cord injured patients. Spinal Cord.

[77] Iwatsubo E, Iwakawa A, Koga H, Imamura A, Yamashita H, Komine S (1985). Functional recovery of the bladder in patients with spinal cord injury: prognosticating programs of an aseptic intermittent catheterization. Acta Urol Japan.

[78] Vaidyanathan S, Soni BM, Brown E, Sett P, Krishnan KR, Bingley J, Markey S (1998). Effect of intermittent urethral catheterization and oxybutynin bladder instillation on urinary continence status and quality of life in a select group of spinal cord injury patients with neuropathic bladder dysfunction. Spinal Cord.

[79] Donnelly J, Hackler RH, Bunts RC (1972). Present urologic status of the World War II paraplegic: 25-year follow-up comparison with status of the 20-year Korean War paraplegic and 5-year Vietnam paraplegic. J Urol.

